# Leicester Royal Infirmary

**Published:** 1920-05-01

**Authors:** 


### LEICESTER ROYAL INFIRMARY.

At a special meeting of the Board of Governors of the Leicester Royal Infirmary (Incorporated by Royal Charter) Mr. T. Fielding Johnson, jun., J.P., was unanimously elected chairman of the Board for the ensuing year. This makes the fifth year during which Mr. Fielding Johnson has presided over the Board.

Progress will be continued during the current year by the building of a new south-west wing, to contain approximately one hundred beds, and by the enlargement of the Nurses' Home, to contain sixty additional bedrooms, in both of which projects the annual meeting readily acquiesced. Mr. C. J. Bon, C.M.G., F.R.C.S., was subsequently elected Vice-Chairman, a position which he has occupied during Mr. Fielding Johnson's chairmanship.

Mr. Alfred Corah's work as Treasurer received warm recognition, and on completion of his three years' term of office his gift of the Institution's Charter was enthusiastically received. In his place the Board appointed Mr. Duncan Henderson, J.P., Treasurer for the ensuing year. Mr. Henderson entered heartily into Mr. Corah's effort for the augmentation of the annual income of the institution, and his assistance was productive of excellent results, which should augur well for the continued financial success of the institution under his treasurership.

The Board subsequently proceeded to the election of an assistant honorary physician, for which appointment there were four candidates, and a ballot resulted in favour of Dr. F. C. Gladstone. Dr. Gladstone was house physician at St. Thomas's Hospital before joining the Navy as temporary surgeon from 1915 to 1919. He was then appointed house physician to the infirmary. He is a Master of Arts of the University of Oxford, having graduated in the Final Honours, School of Physiology, and he is a Doctor of Medicine and a Bachelor of Surgery of the same University.

